# A community-based cluster randomized controlled trial (cRCT) to evaluate the impact and operational assessment of “safe motherhood and newborn health promotion package”: study protocol

**DOI:** 10.1186/s12889-018-5478-6

**Published:** 2018-05-03

**Authors:** Dewan Md. Emdadul Hoque, Mohiuddin Ahsanul Kabir Chowdhury, Ahmed Ehsanur Rahman, Sk. Masum Billah, Sanwarul Bari, Tazeen Tahsina, Mohammad Mehedi Hasan, Sajia Islam, Tajul Islam, Rintaro Mori, Shams El Arifeen

**Affiliations:** 1 Maternal and Child Health Division, International Centre for Diarrhoeal Diseases Research, Bangladesh (icddr,b), 68, Shahid Tajuddin Sarani, Mohakhali, Dhaka, 1212 Bangladesh; 2 MaMoni Health Systems Strengthening Project, Save the Children, Dhaka, Bangladesh; 3 National Centre for Child Health and Development, 2-10-1 Okura, Setagaya-Ku, Tokyo, 157-8535 Japan

**Keywords:** Community, Cluster randomized controlled trial, Safe motherhood, Newborn survival

## Abstract

**Background:**

Despite considerable progress in reduction of both under-five and maternal mortality in recent decades, Bangladesh is still one of the low and middle income countries with high burden of maternal and neonatal mortality. The primary objective of the current study is to measure the impact of a comprehensive package of interventions on maternal and neonatal mortality. In addition, changes in coverage, quality and utilization of maternal and newborn health (MNH) services, social capital, and cost effectiveness of the interventions will be measured.

**Methods:**

A community-based, cluster randomized controlled trial design will be adopted and implemented in 30 unions of three sub-districts of Chandpur district of Bangladesh. Every union, the lowest administrative unit of the local government with population of around 20,000–30,000, will be considered a cluster. Based on the baseline estimates, 15 clusters will be paired for random assignment as intervention and comparison clusters.

The primary outcome measure is neonatal mortality, and secondary outcomes are coverage of key interventions like ANC, PNC, facility and skilled provider delivery. Baseline, midterm and endline household survey will be conducted to assess the key coverage of interventions. Health facility assessment surveys will be conducted periodically to assess facility readiness and utilization of MNH services in the participating health facilities.

**Discussion:**

The current study is expected to provide essential strong evidences on the impact of a comprehensive package of interventions to the Bangladesh government, and other developmental partners. The study results may help in prioritizing, planning, and scaling-up of Safe Motherhood Promotional interventions in other geographical areas of Bangladesh as well as to inform other developing countries of similar settings.

**Trial registration:**

NCT03032276.

## Background

An estimated 3.1 million neonatal deaths take place worldwide, representing two-thirds of all infant deaths [1, 2]. Almost half of these neonates die on the first day of life [2, 3], and three fourths within the first 7 days [4]. Seventy percent of these deaths occur in low income countries of Africa and Asia [5] where the majority of the world’s poor population live. Two thirds of global child deaths may be prevented by available and affordable interventions that are feasible for high population coverage in low income countries [6]. On the other hand, despite the achievement of halving maternal mortality over the past two and a half decades, 289,000 women die globally each year as a consequence of pregnancy and child birth [7]. In 2015, 830 women died daily due to complications of pregnancy and child birth [7]. Annually, 189 million women become pregnant, 10% having complications during pregnancy or in the intrapartum period [8]. Moreover, 40% of recently delivered women live with post-delivery morbidities or disabilities [7]. Maternal deaths during pregnancy and child birth are more prominent in low and middle income countries, most of which may be prevented by utilization of evidence based low cost interventions [8]. The risk of death from a maternal-related cause is 33 times higher for women in developing countries compared to women in developed countries [9]. The primary causes of these deaths are haemorrhage, hypertension, infections, and indirect causes, mostly due to interaction between pre-existing medical conditions and pregnancy [1, 9]. Such statistics highlight the importance of focusing on perinatal and early postnatal periods to prevent maternal and neonatal mortalities. In this context, to foster worldwide progress, the World Health Organization (WHO) in the General Assembly, adopted the resolution of sustainable development goals, which include the agenda to reduce both maternal and neonatal mortality rates [10].

Bangladesh has shown considerable progress in the reduction of child and maternal mortality in recent decades [11, 12]. From 1994 to 2014, under-5 mortality rate has reduced by about 65% from 133 to 46 per 1000 live births, while neonatal mortality declined by 46% from 52 to 28 per 1000 live births. Currently, neonatal mortality accounts for 74% of infant deaths and 61% in children aged below 5 years [13]. Bangladesh has seen a 70% decline of Maternal Mortality Ratio (MMR) between 1990 and 2013, from 574 to 170 per 100,000 live births, with an annual a reduction rate of 5.0% [11]. In line with global commitments, Bangladesh renewed targets to reduce neonatal mortality to 12 per 1000 live births and maternal deaths to below 70 per 100,000 live births by 2030 [2, 14]. The challenge for the future is to sustain and accelerate the pace of reduction of maternal and newborn mortality.

### Safe motherhood promotion project (SMPP) in Bangladesh

In Bangladesh, successive governments have continued to prioritize health, and take alternative measures to create awareness in communities [15]. Reasons for under-use of existing health services are complex, and in order to increase uptake, it is important that physical access barriers are removed. In addition, service quality and community perceptions of service providers need to be addressed. The success of Millennium Development Goals and immunization have largely been dependent upon extensive involvement of community level health care providers which include Health Workers, Health Assistants, Family Welfare Assistants), Health Inspectors), Sub-Assistant Community Medical Officers and other health professionals. To overcome the problems of serving the community, the Government of Bangladesh (GoB) established 13,500 community clinics, run by Community Health Care Providers (CHCP), to provide a comprehensive set of maternal, newborn, child health (MNCH) and family planning services. Community groups (CG) are the key component of the community clinic initiative, being the principal driving force behind the organization, management, maintenance, and quality of care provided at the community clinics. In 2006, a five year pilot project concentrating around the community clinics was launched by the GoB in collaboration with Japan International Cooperation Agency, with an aim to improve the health status of pregnant and postpartum women and neonates. An intervention package was implemented through three linked activities: advocacy at the central level, strengthening of health facilities, and empowerment of community. To improve service delivery, a Hospital Improvement cycle (‘plan-do-see’ process) for Emergency Obstetric Care services (EmOC) was undertaken at selected public hospitals. The union level intervention was called the Model Union approach, comprised a package of activities involving community level health care providers, community stakeholders, local government bodies, community people and so forth. SMPP was successful in empowering the community to tackle Maternal Newborn Health issues through the development of Community Support System, a system to provide support to pregnant women and newborns during obstetric emergencies through organizing the community people. This activity was facilitated by CARE Bangladesh, in three sub-districts of Narsingdi. Introduction of Private Community Based Skilled Birth Attendants (CSBA) in remote areas was another initiative taken by SMPP, in collaboration with the local government [16, 17].

Evaluation of SMPP revealed positive changes after implementation of interventions: improvement in United Nation process indicators on EmOC including met needs (increase from 31% to 55%), case fatality rate (< 0.1%), and skilled birth attendance (decline from 18% to 25%). Substantial increases from baseline were observed in: any ANC (55% vs. 73%) / PNC (14% vs. 23%) rates in the model unions, institutional deliveries (14% vs. 21%), women’s birth planning knowledge (44% vs. 93%), at least three danger signs during pregnancy (3% vs. 38%), and linkages between service providers and users. Qualitative findings showed that the service providers’ behaviour had improved and become more client oriented, and that mothers had increased satisfaction and trust in the public service providers. Some of the identified bottlenecks were a shortage of human resources, frequent transfers of trained personnel, insufficient budget, and lack of supportive supervision. SMPP was recognized as a model MNH initiative and called the “Narsingdi Model” at the 2010 Bangladesh Development Forum and 2010 G8 summit [16].

### Rationale of the current study

Despite successes depicted by the evaluation of SMPP, the results were inconclusive due to the unavailability of concurrent comparison areas. Therefore, this current project will conduct a rigorous evaluation of the newly designed programme titled ‘Safe Motherhood Promotion and Newborn Survival (SMPNS)’ using a cluster Randomized Controlled Trial (cRCT) design. The objective is to assess and measure the effect of an intervention package, when implemented at scale, in improving maternal and neonatal health. It is important to: (i) monitor and evaluate performance and impact of the approach; (ii) document the implementation process, identify barriers and facilitators of the implementation of SMPNS components for suggesting probable solution(s); (iii) describe non-health impacts of SMPNS; (iv) address these barriers and utilize the facilitators; and (v) conduct cost- effectiveness analysis. The intervention package is expected to raise awareness, increase social mobility and interactions, and contribute to a more stable social safety net. Consequently, the proposed approach should have a potential to increase social capital in a community, thereby reduce harmful health outcomes. Though social capital has received some research focus since the 1990s, it still remains under-theorized. This will be an important avenue to explore and contribute to as part of this project. We expect that the results will provide essential and policy-relevant findings on programme strategies to be implemented at scale, to improve maternal and newborn health in the rural areas of Bangladesh. The findings will help the Ministry of Health and Family Welfare (MOHFW) of the BG and development partners in prioritizing, planning, and further scaling-up of SMPP in other areas of Bangladesh but will also help inform other developing countries in similar settings.

## Methods

### Description of interventions

**Fig. 1:**
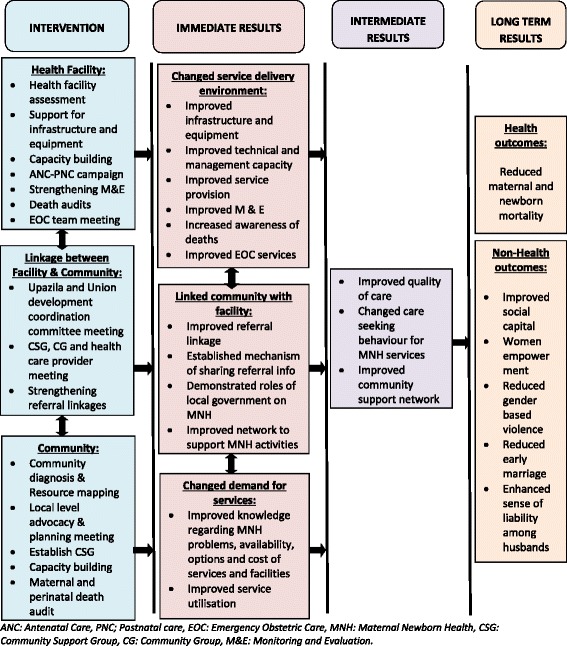
Conceptual Framework

The intervention package consists of three components: facility, community and linkages between the community and health facilities. Facility based interventions will serve both the comparison and intervention arms. Community based interventions related to promotion of community linkage will serve as comparison. Specific interventions and approaches will be implemented within the MOHFW service delivery system to improve MNH practices in families and communities and to increase the use of MNH services from appropriate facilities/providers. The intervention package (Table 1) has been developed based on the experiences from a previous SMPP model and Fig. 1: Conceptual framework describing interventions, short term and long term outcomes.

A. Health facility interventions

**Table 1:** Interventions at health facility and Community levels and linkage

	Interventions	Comparison Arm	Intervention Arm
Facility	District Hospital	Health Facility Assessment and planning Resource support for infrastructure and equipment Capacity building ANC and PNC campaign Strengthening monitoring and supportive supervision Perinatal death audits in facilities EOC team meeting	
	Upazila Health Complex		
	Union Sub-Centre and Family Welfare Centre		
Linkage	Union and Upazila development coordination committee meeting	As per the GoB system	Facilitation
	CSG, CG and health provider meeting	As per the GoB system	Facilitation
	Strengthening referral linkages through active facilitation	As per the GoB system	Facilitation
	Observe relevant days	No	Yes
Community	Community diagnosis and resource mapping	As per the GoB system	Active Facilitation through UH&FPO
	Local level advocacy and planning meeting	As per the GoB system	Training and orientation
	Establish CSG	As per the GoB system	Active Facilitation through Union Parishad
	Capacity building of CG and CSG members	No	Yes
	Maternal and perinatal death audit in the community	No	Yes
	Creating enabling environment for women and children	As per the GoB policy	Active Facilitation
	Community death audits	No	Yes

*UH&FPO* Upazila Health & Family Planning Officer, *CSG* Community Support Group, *CG* Community Group, *GOB* Government of Bangladesh, *ANC* Antenatal Care, *PNC* Postnatal care, *EOC* Emergency Obstetric Care

Adequate efforts will be made to improve the provision and quality of MNH services in the targeted health facilities. The possible increase in demand for services, as a result of the intensive community mobilization activities, and improved quality of care in the facilities, will also be addressed. The facility interventions (Table 1) will be carried out in selected public facilities in both intervention and comparison unions. The targeted facilities will include the Chandpur District Hospital, Maternal & Child Welfare Centre, Upazila Health Complexes, Union Sub-Centres, Union Health and Family Welfare Centre, and Community Clinics. The activities for health facility interventions will include:

1. Health facility assessment and planning.
2. Resource support for improving infrastructure and equipment.
3. Capacity building.
4. Anti Natal Care, Post Natal Care campaign.
5. Strengthening monitoring and supportive supervision.
6. Service facilitation through welcoming person.
7. Maternal and neonatal death review.
8. Emergency obstetric care team meeting.

B. Linkage between facilities and community

It is expected that through the facilitated implementation of facility and community interventions, the demand for services and responsiveness of service providers will be improved. Development of a functional partnership between facilities and communities will lead to sustainable improvements from the interventions; activities for improving partnership will include:

1. Union and Upazila Development Coordination Committee meetings.
2. CSG and health providers’ meetings.
3. Strengthen referral linkages.
4. Observe relevant days.

C. Community interventions

Community interventions will enhance MNH-related knowledge of families and communities, and improve their MNH-related practices, by increased service utilisation. Effective community activities (ref) include:

1. Community diagnosis and resource mapping exercise
2. Local level advocacy and planning meeting
3. Establish Community Support Group (CSG)
4. Capacity building of CSG members, key community gate keepers and Union Parishad representatives for implementing
5. Promoting birth planning, ANC, PNC and ENC counselling sessions through engaging selective CSG members, community volunteers, MOH&FW and NGO workers
6. Maternal and perinatal death audit at community level
7. Creating an enabling environment for women and children through relevant community stakeholders

### Study design

A community-based, cRCT design will be used for the evaluation. The union, the smallest administrative unit in Bangladesh, will be considered as a cluster, i.e., the unit of randomisation. Both quantitative and qualitative approaches will be adopted to address general and specific objectives.

### Study setting

**Fig. 2:**
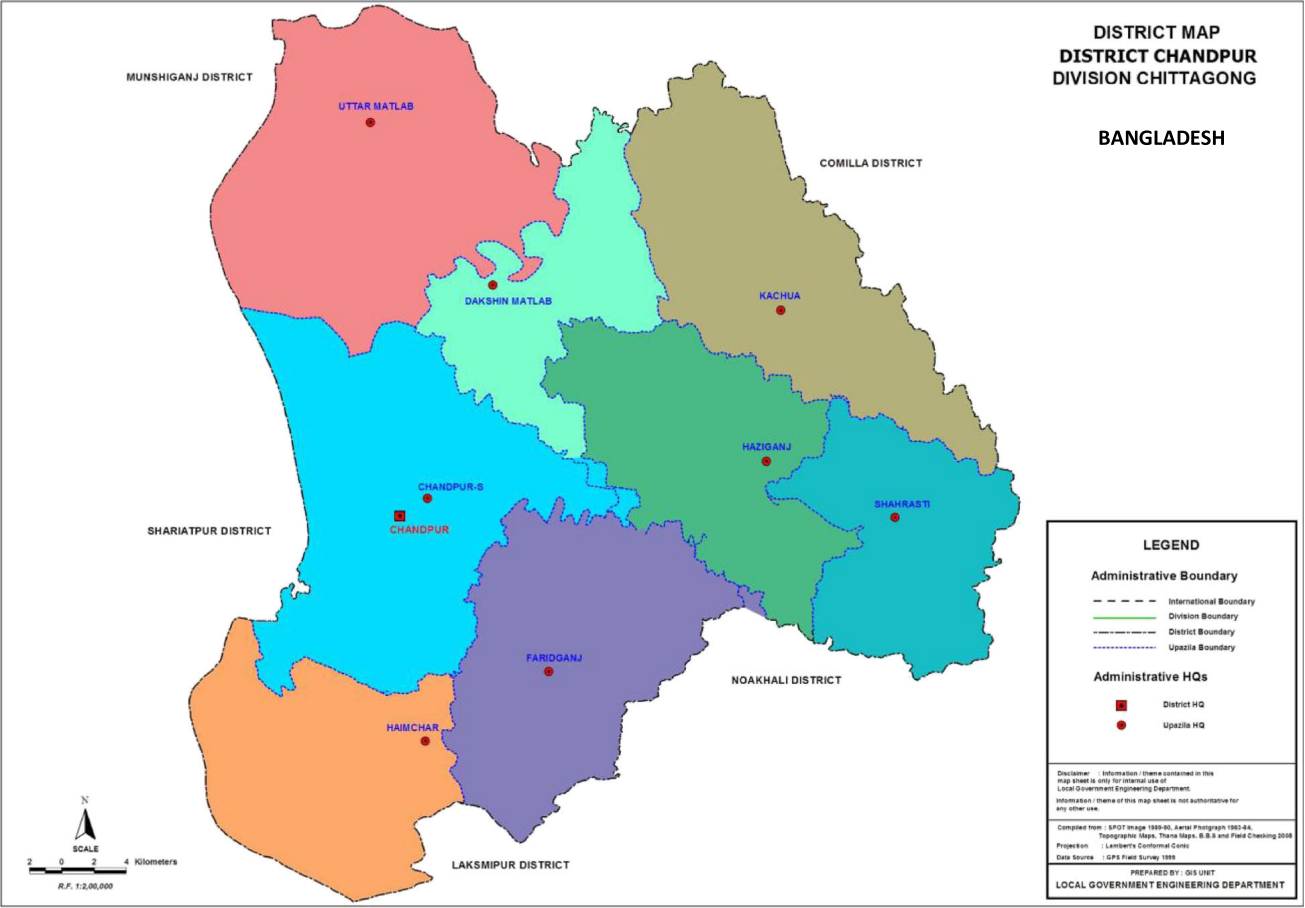
Study location

We proposed to conduct the study in Chandpur district. The basic geography and demography of Chandpur district are provided in Table 2 and the map is shown in Fig. 2: Map showing study location.

**Table 2:** Basic geography, urban and rural population of Chandpur District, Bangladesh

Area (sq km)	Upazila	Municipality	Union	Mouza	Village	Population	
						Urban	Rural
1704.06	8	6	87	1062	1237	314,102	1,957,127

Source: http://www.lged.gov.bd/DistrictHome.aspx?districtID=12

While the GoB has implemented interventions models across Bangladesh, none have been implemented in Chandpur. Though the district level maternal and child health indicators of Chandpur are comparable with national estimates, the sub-districts significantly lack behind. Three upazilas have been selected for intervention implementation, namely Chandpur Sadar, Faridganj, and Kachua as their estimates are below the national estimates. Based on baseline estimates, pairing will be done among selected 30 unions. During pairing, the availability of health facilities and services, demographic and socioeconomic indicators, contextual factors (presence of other significant programmes/initiatives), and population size will be taken into account. Unions from each pair will be randomly allocated to either intervention (n = 15) or comparison (*n* = 15) arm.

### Selection of unions and randomisation:

Step 1 - selection of eligible unions: Contextual information for all unions of the three selected upazilas will be collected to include:

- Number of Community Skilled Birth Attendants (CSBA)
- Ongoing or planned maternal and child health services / programmes by BG or NGO. This information will be obtained from relevant government programme managers, upazila managers and NGO managers
- Number of functional community clinics
- Terrain (proportion of wetlands, hill, forest), accessibility (average time to reach union health facility from the farthest point within union, and approximate travel time to reach UHC from union health facility). This information will be obtained from facility service providers attending UHC monthly meetings.
- Number of active NGOs and type of NGO activities

If a union has a total population of less than 12,000 (the minimum required to obtain an adequate neonate sample they will either be excluded or merged with another smaller union if exclusion results in fewer than 30 unions.

Step 2 - Principal Component Analysis (PCA) and pairing: A PCA score, for each union, will be developed using the following indicators.

- Demographic and socio-economic information from Bangladesh Bureau of Statistics, on number of villages, households, population, population density, literacy rate, school attendance, proportion of persons with no work, percentage of households drinking safe water and using sanitary toilets, proportion of households with electricity, and proportion of households receiving remittance income. This will then be followed by the final step where contextual and available facilities will be taken into account to derive the final set of pairs for the trial.
- Type of union level facilities and functionality will be collected as part of the upazila selection process.

Fifteen pairs will be constructed based on the PCA scores (closest match).

Step 3 – Randomisation: From each of the 15 union pairs, one union will be randomly allocated to the intervention arm, and the other to the comparison arm.

### Study population

The intervention (15) and comparison (15) union areas will cover approximately a total population of 375,000, in 90,000 households.

### Sample size

Sample size was estimated using standard pair randomized controlled trial sample size calculation formula (ref). Using the Chandpur specific neonatal mortality estimate (20 per 1000 live births) [18], it has been estimated that 26 clusters are required (13 each in intervention and comparison arm, using a k = 0.1) to detect a minimum 25% reduction in neonatal mortality, assuming a refusal rate of 5%. The number of clusters in both intervention and comparison arms were fixed at 15, with consideration of occurrences of unexpected events for 1–2 clusters. The required cluster size/number of children/RDW (n) for a given indicator, for each study arm, was estimated to be X. The estimation was completed iteratively with α level of 95% (two tail), and 80% power.

An estimated sample of 1050 live births will be required for each union, to measure a 15% decrease of neonatal deaths from 20 to 15 per 1000 live births between baseline and endline. In order to measure the knowledge, practice, and health seeking behaviour of the community to MNH issues, the following numbers of respondents from each cluster is required to be interviewed to detect the desired effect size with 80% power:

1. Live birth within two months of survey: 35.
2. Live births at least one danger sign during the neonatal period within two months of survey: 30.
3. Recently delivered women within 12 months of survey: 525.
4. Recently delivered women with at least one danger sign during pregnancy within 12 months of survey: 40.

### Data collection methods

Data collection will include household surveys at baseline, two midlines and endline, to measure the impact of interventions on neonatal and maternal mortalities. Two midline surveys will be conducted on a sub set population. Health facility assessments surveys will be conducted to assess the availability and quality of MNH services in the participating health facilities. Relevant data from routine Health Management Information System (HMIS) will be regularly collected and extracted from hospital records to monitor the utilization trend in the health facilities. An ongoing investigating system will be in place at the health facilities, to identify and document maternal near miss cases. Qualitative data will be collected for process documentation, and in depth exploration of implementation challenges and contextual factors. Both quantitative and qualitative methods will be employed to measure social capital. Qualitative data collection methods will include stakeholders’ meeting, focus group discussions, periodic in-depth assessments, social mapping, and other participatory exercises. These standard methods will be employed to document effects of the intervention on women’s empowerment, as measured by decision-making and mobility, within the intervention communities. Table 3 summarizes the evaluation plan of the study.

**Table 3:** Project aims, indicators, expected outcomes and tools for evaluation of the study

Aims	Expected outcomes	Indicators	Tools
To measure the effectiveness of intervention on neonatal mortality.	Improved survival of neonates and improved MNH in the community.	Neonatal mortality rate.	Household survey, death audits, and Management Information System (MIS).
To assess the effect of the intervention on knowledge and practices related to MNH at community.	Improved MNH knowledge and practices of participants (mothers / caregivers / families at home).	Proportion of Recently Delivered Women (RDW) having knowledge of danger signs; delivered by skilled birth attendants; delivered at facility; received at least 4 ANC and PNC within 2 days. Proportion of newborn received Essential Newborn Care.	Household survey, and qualitative assessment.
To evaluate the effect of intervention on health and care seeking behaviour.	Increased knowledge of participants which translates into appropriate care-seeking behaviour.	Proportion of RDW who sought care from trained providers for complications. Proportion of neonates that were taken to trained providers with at least one danger sign.	Household survey, MIS, and qualitative assessment.
To measure and compare utilization of facilities for neonatal illness episodes and complications.	Increased, sustained utilization of facilities for neonatal and maternal cases.	Number of neonatal and maternal cases managed at health facilities. Number of maternal near miss cases managed at health facilities.	MIS.
To measure and compare the quality of care for neonates and mothers.	The quality of care at different level of facilities will be increased.	Facility readiness to provide quality care in terms of infrastructures, staffs, trainings, and logistics.	Assessment of health facility and, quality of care, and qualitative assessment;
To measure incremental costs and estimate the cost-effectiveness.	Costs for improved neonatal health outcomes, and related health services.	Costs per Disability Adjusted Life in Years (DALY) averted, costs per life-year gained, costs per case of delivery by skilled attendance, costs per case of essential neonatal care, programme cost, incremental health service costs for providing quality services, and out of pocket expenditure.	Client interview at hospitals, facility records, semi-structured interviews, and review of programme spending

*MIS* Management Information System, *MNH* Maternal and Newborn Health, *ANC* Antenatal Care, *PNC* Postnatal care

### Data collection procedures, data quality and monitoring

Before participant recruitment, the data collection team will be trained by the central team on all aspects of data collection, including consent, data collection methods, questioning techniques, and cultural considerations. Refresher training will be conducted as required. Data collectors will be supervised by Field Research Assistants who will monitor data collection quality, and provide on-site feedback. A field manager will be responsible for overall project coordination. There will be separate data collection teams for household listing, household survey, health facility assessment, qualitative data collection, and social capital measurement.

## Duration of the project

We started collecting baseline data for randomisation and selection of intervention and comparison clusters. We anticipate that selection of non-government organization to facilitate the interventions will commence in January 2018. Randomisation will be completed by March 2018 and intervention will be started to roll out in April 2018. Details implementation timeline including implementation of intervention in comparison clusters are illustrated in Table 4.

**Table 4:**
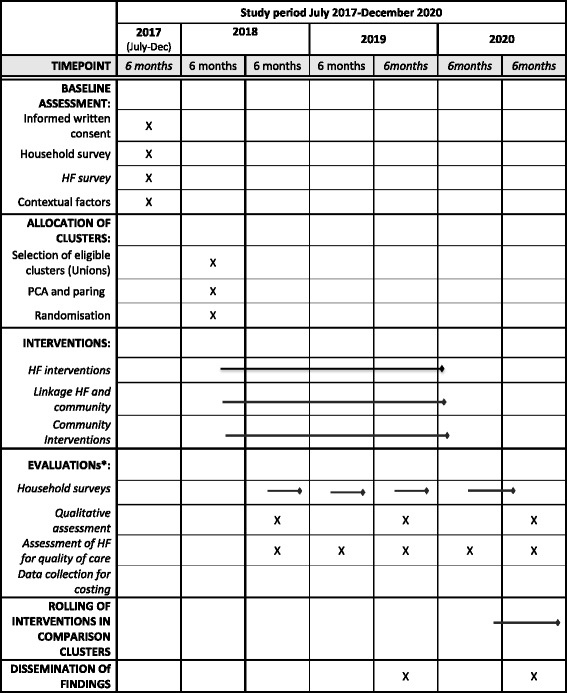
Project duration and timeline for baseline assessment, randomization, Interventions and evaluation of the study

^a^Descriptions of evaluation plan in Table 3; *HF* Health Facility, *PCA* Principal Component Analysis

### Trial outcomes and measurements

Trial outcomes and measurements are summarized in Table 3.

Primary outcomes are improved survival of neonates and improved MNH in the community.

Secondary outcomes include improved MNH related knowledge, practices and care seeking behaviour of community people, increased and sustained utilization of facilities for neonatal and maternal cases, improved quality of health care, social capital of the community people, and measured costs.

Other measurements include health care seeking behaviour during maternal and neonatal emergencies and household coverage of MNH interventions with knowledge regarding MNH issues and utilization of relevant services.

### Data management plan

All data obtained from the field will be entered in databases using Microsoft SQL Server 2005 with Visual Basic 6.0 for the user interface. This will be a Relational Database Management System (RDMS) where all data tables will be linked with unique primary and secondary keys. Range and consistency checks will be in-built to prevent errors during data entry. The learning platform will use MIS data from the health facilities, using information from the ongoing household surveillance, to measure maternal severe morbidity (near miss) and case-fatality rate for EmOC.

### Data analysis plan

Data will be analysed by investigators under the guidance of the expert panel, to maintain accuracy and validity. Descriptive statistics will be utilized as appropriate to describe the baseline and socioeconomic characteristics of the respondents, and facility level management. The household surveys will provide a period-specific rate for the various indicators, and information on severe maternal complications, care seeking behaviour, and neonatal and maternal deaths. Socio-demographic information collected within the survey will be used for equity analysis. Bi-variate and multivariate analysis will be used to assess the determinants of the various primary and secondary outcomes from both household and facility level surveys. Potential explanatory variables estimating relative effects after adjusting for confounder will be included. Equity analysis will be conducted for all indicators. For equity analysis, we created one absolute indicator of inequality (the slope index of inequality), and two relative inequality indicators (ratio of Q5 to Q1, and the concentration index). The cost-effectiveness analysis will consider costs per Disability-Adjusted Life Years (DALYs) averted, per life-year gained, costs for delivery by skilled attendance, essential newborn care, and out of pocket payment for care seeking for maternal and newborn complications. Intervention costs will be estimated based on WHO-CHIOICE ingredients approach, and include programme and patient costs [19]. Programme costs will be estimated through cost records, activity-based costing, and facility financial data. Incremental cost for delivering the intervention will be measured through the household surveys. These costs include any direct financial payments for services, transportation, and time spent using the services. Based upon the expected neonatal and health service improvement outcomes, the decisional simulation model will be developed to measure cost-effectiveness of the proposed comprehensive package. To test the robustness of the model, sensitivity analysis will be performed for uncertainties regarding quality, and performance and utilization of maternal and child health services, which potentially affect cost-effectiveness. All the quantitative analyses will be adjusted for probability sample design. STATA 13 will be used for all household and facility level statistical analysis, and Tree Age Pro for cost-effectiveness analysis.

Interviews, focused discussions, and field notes from all qualitative data collection methods, will be recorded and transcribed verbatim. Upon preparing the transcriptions, data will be coded by the researchers. To establish inter-coder reliability, each researcher will code a minimum set of interviews. After which, a master code-list will be generated, based on which the rest of the transcripts will be coded. Future additions and changes to the code-list will be duly communicated to all the researchers. The next step in analysis will be to generate summaries of codes and analyse based on a theoretical phenomenological orientation. The social capital will be measured in structural and cognitive dimensions. We hypothesize that the interventions will connect people and strengthen contacts, thereby increase knowledge of available resources, improve ability to access health services, and adopt beneficial health-related practices. Cognitive social capital, measures the degree to which people perceive they have support from others in the network, can trust others, and seek their help in times of need. We assume that the interventions will create and sustain relationships of trust and support, and this in turn will result in improvements in health. Measurement instruments will be adapted from the World Bank’s Social Capital Assessment Tools developed to assess social capital in the developing nations [20], to be contextually relevant and culturally sensitive. It will be designed to assess both structural and cognitive social capital, and easily understandable to both interviewers and informants. Individual level measures will be analysed to assess the effects of the intervention on access to social capital among community members, as a potential mediator of individual health effects.

### Project governance

An Implementation Committee (IC) will be formed to oversee project implementation and procurements required related to the study. A Steering Committee (SC), chaired by a representative from Ministry of Health and Family Welfare, BG, will be formed to meet every semester, to monitor project progression, and guide activities in adherence to objectives and goals.

For conducting research related activities, six teams comprising young and mid-level researchers, will be formed. The teams will meet weekly to update the project director and project co-ordinator of progress. The teams will be employed for the following thematic areas of assessment: i) baseline, midline and endline survey; ii) facility; iii) social capital; iv) cost-effectiveness; v) Qualitative data collection; and vi) process documentation;

### Dissemination plan

At the completion of the endline survey, a dissemination workshop will be held to inform the local and national relevant stakeholders. In the event of positive outcomes, a policy brief will be prepared to guide policy makers to move forward with the intervention package. All findings will be presented in international scientific conferences, and submitted for peer-reviewed publication.

## Discussion

The current study is expected to provide essential strong evidences on the impact of a comprehensive package of interventions to the Bangladesh government, and other developmental partners. The Government of Bangladesh will lead the study collaborative partners which include icddr,b, University of Tokyo, and implementing partner NGO. To maintain proper communication among the partners and execute the decision on time might be proven to be a challenge for this study. To mitigate this obstacle, a monthly call will be arranged among the partners and the minutes would be documented accordingly. One of the noteworthy strengths of the current study is the measurement of social capital which is comparatively less explored area in the context of Bangladesh. The social capital assessment in this SMPNS study would inform the scientific world a novel information regarding its association with care seeking behaviour, utilization of public health facility and ultimately with mortality indicators. Another interesting component of the study is community engagement. The study involves the community actively through CG and CSG which is one of the core essences of the community based interventions. This community based interventions may bring forth revolutionary changes for sustainable improvement in MNH sector. The study will also evaluate the impact of interventions for linkage between facility and community that may reduce the delays and ultimately affect the mortality rates. It is noteworthy to mention again that the facility based interventions will be implied on both comparison and intervention unions which would help to improve quality of care provided in the public health facilities and also will deal with the ethical issues, if there is any. This approach would help to understand the relationship between quality of care and care seeking behaviour of the community people. Moreover, the impact of improved quality on mortality rates can also be estimated from the current study. We expect to perform rigorous evaluation of the intervention maintaining utmost quality all through the project starting from data collection through final data analysis and dissemination of results. The study results may help in prioritizing, planning, and scaling-up of Safe Motherhood Promotional interventions in other geographical areas of Bangladesh as well as to inform other developing countries of similar settings.

## Abbreviations

ANC: Ante Natal Care
CG: Community Group
CHCP: Community Health Care Provider
cRCT: Cluster Randomized Controlled Trial
CSG: Community Support Group
EmOC: Emergency Obstetric Care
GoB: Government of Bangladesh
MMR: Maternal Mortality Ratio
MNCH: Maternal, Newborn and Child Health
MNH: Maternal and Newborn Health
MOHFW: Ministry of Health and Family Welfare
NGO: Non-Government Organization
PCA: Principal Component Analysis
PNC: Post Natal care
SMPNS: Safe Motherhood Promotion and Newborn Survival
SMPP: Safe Motherhood Promotion Project
WHO: World Health Organization
